# Other PHAC publications

**DOI:** 10.24095/hpcdp.46.5.05

**Published:** 2026-05

**Researchers from the Public Health Agency of Canada also contribute to work published in other journals and books. Look for the following articles published in 2026:**

**Ahmad R, Kakkar T, Rotondo J, Hamilton K**, Bowes MJ, Jones G, **Leung Soo C, VanSteelandt A**. Substances and substance combinations among accidental substance-related acute toxicity deaths (AATDs) in Canada from 2016 to 2017. BMC Public Health. 2026;26(1):90. https://doi.org/10.1186/s12889-025-22777-2

Baidoobonso S, Clark EC, Noonan LL, Bakker J, **May-Hadford J**, Phillips KA, et al. Mobilizing community-led health promotion: evidence-informed co-development of the Live Well PEI community mobilization platform and integrated granting program. Can J Public Health. 2026. https://doi.org/10.17269/s41997-025-01140-3

Beauchamp MK, D’Amore C, Raina P, McIlroy W, Adesina N, Ahmadi M, […] **Prince SA**, et al. Establishing global standards on wearable technology for measuring mobility in ageing populations: an international consensus exercise. Age Ageing. 2026;55(1):afaf376. https://doi.org/10.1093/ageing/afaf376

Choi SM, Dong H, Lei SM, Tomkinson GR, **Lang JJ**, Cadenas-Sanchez C, et al. A 15-year decline in physical fitness among children and adolescents from the Macao Special Administrative Region (2005–2020). J Phys Act Health. 2026;23(2):254-62. https://doi.org/10.1123/jpah.2025-0532

Halsall T, **Orpana H**, Jan M. Tracing the undercurrents: a scoping review of the lifestyle drift concept. BMC Public Health. 2026; 26(1):321. https://doi.org/10.1186/s12889-025-25616-6

**Lau E, Plouffe R, Liu L, Contreras G, Johnson K, Garipy G**. Seasonal variations in suicide mortality in Canada: a nationwide analysis. Can J Public Health. 2026. https://doi.org/10.17269/s41997-026-01155-4

**Prince SA, Thomas T**, Biswas A. The effects of occupational and leisure time physical activity on health-related quality of life: a repeated-measures longitudinal study. Sports Med. 2026. https://doi.org/10.1007/s40279-025-02382-4

Tollenaar SL, Khorasaniha R, Jovel J, Ba I, Voisin A, Miller R, […] **Bonner C**, […] **Graham M**, et al. Reduced fibre-fermenting capacity of gut microbes in multiple sclerosis may result in prebiotic dietary fibre β-fructan promoting inflammation and CNS damage. eGastroenterology. 2026;4(1):e100296. https://doi.org/10.1136/egastro-2025-100296

